# Radiation-induced YAP activation confers glioma radioresistance via promoting FGF2 transcription and DNA damage repair

**DOI:** 10.1038/s41388-021-01878-3

**Published:** 2021-06-14

**Authors:** Yu Zhang, Yan Wang, Ding Zhou, Kai Wang, Xu Wang, Xiang Wang, Yang Jiang, Min Zhao, Rutong Yu, Xiuping Zhou

**Affiliations:** 1 Institute of Nervous System Diseases, Xuzhou Medical University, Xuzhou, Jiangsu China; 2 Department of Neurosurgery, The Affiliated Hospital of Xuzhou Medical University, Xuzhou, Jiangsu China; 3 The Graduate School, Xuzhou Medical University, Xuzhou, Jiangsu China

**Keywords:** CNS cancer, Cell signalling

## Abstract

Although radiotherapy is a well-known effective non-surgical treatment for malignant gliomas, the therapeutic efficacy is severely limited due to the radioresistance of tumor cells. Previously, we demonstrated that Yes-associated protein (YAP) promotes glioma malignant progression. However, whether YAP plays a role in radioresistance and its potential value in cancer treatment are still unclear. In this study, we found that high YAP expression is associated with poor prognosis in malignant glioma patients undergoing radiotherapy. Research in immortalized cell lines and primary cells from GBM patients revealed that YAP exhibited a radioresistant effect on gliomas via promoting DNA damage repair. Mechanistically, after radiation, YAP was translocated into the nucleus, where it promoted the expression and secretion of FGF2, leading to MAPK–ERK pathway activation. *FGF2* is a novel target gene of YAP. Inhibition of YAP–FGF2–MAPK signaling sensitizes gliomas to radiotherapy and prolongs the survival of intracranial cell-derived and patient-derived xenograft models. These results suggest that YAP–FGF2–MAPK is a key mechanism of radioresistance and is an actionable target for improving radiotherapy efficacy.

## Introduction

Malignant glioma is the most common primary intracranial tumor and has a very short average survival time [1, 2]. Radiotherapy, in which cancer cells are killed by inducing DNA damage beyond the cellular capacity to repair, is considered to be the most effective non-surgical treatment for malignant glioma. However, therapeutic efficacy is severely limited due to the high intrinsic radioresistance of glioma cells [3].

Glioma radioresistance has numerous molecular bases [3] and any factors favoring DNA repair efficiency would cause resistance to therapies relying on DNA damage. For example, hyperactivation of the AKT pathway is associated with the radioresistance effect of glioma by promoting DNA damage repair [4]. Nuclear phosphatase and tensin homolog (PTEN) tyrosine phosphorylation confers radioresistance to glioma cells and inhibition of it enhances radiation sensitivity through attenuating DNA repair [5]. Sonic hedgehog (shh) pathway activation in glioma stem cells (GSCs) enhances non-homologous end joining-mediated repair of DNA damage, promoting cell resistance to radiation [6], whereas inhibition of the shh pathway enhances the radiosensitivity of GSCs [6]. However, the molecular mechanism of radioresistance of gliomas is largely unknown. Therefore, it is urgent to understand the molecular mechanism of radioresistance, design novel strategies to sensitize glioma cells to radiotherapy, and improve the prognosis of glioma patients.

The Hippo pathway plays a substantial role in organ size control, cell growth, and apoptosis [7]. As a key effector of the Hippo pathway, Yes-associated protein (YAP) functions as a transcription co-activator [8], which binds to TEA domain (TEAD) family members and then activates its target genes, such as *Cyr61*, *CTGF*, and *Axl* [9]. Accumulating evidence suggests that elevated YAP expression or nucleus enrichment has been found in many human tumors, such as liver and breast tumors [10–12]. Our systematic studies identified that YAP is significantly upregulated in gliomas, contributing to glioma cell migration and invasion [13]. In addition, YAP promotes human glioma growth through inhibiting GSK3β and subsequently activating Wnt/β-catenin signaling [14]. Interestingly, several studies have demonstrated that YAP activation is involved in resistance to anticancer therapy in various tumors in recent years [15]. Downregulation of YAP in urothelial cell carcinoma promotes DNA damage and apoptosis after radiation [16]. In medulloblastoma, inhibition of YAP permits reduction of the radiation dose required to induce tumor cell death [17]. However, the molecular mechanism of the effects of YAP on radioresistance and its potential value in cancer treatment is still unclear.

Here we show that high YAP expression suggests poor prognosis for glioma patients with radiotherapy and radiation activates YAP, which contributes to glioma progression after radiation via driving the expression of fibroblast growth factor 2 (FGF2) and subsequently activating the mitogen-activated protein kinase (MAPK) pathway. YAP–FGF2–MAPK pathway activation endows glioma cells with the ability to enhance DNA repair, boost the cell cycle, and inhibit apoptosis, leading to cell survival after radiation. Inhibition of YAP–FGF2–MAPK sensitizes gliomas to radiotherapy. Our novel findings clarify a link between oncogenic YAP and radioresistance, suggesting that the inhibitors of the YAP–FGF2–MAPK pathway may have therapeutic value for patients with high YAP expression by restoring radiosensitivity and inducing glioma cell death after radiation.

## Results

### High YAP expression suggests poor prognosis in glioma patients undergoing radiotherapy

**Fig. 1:**
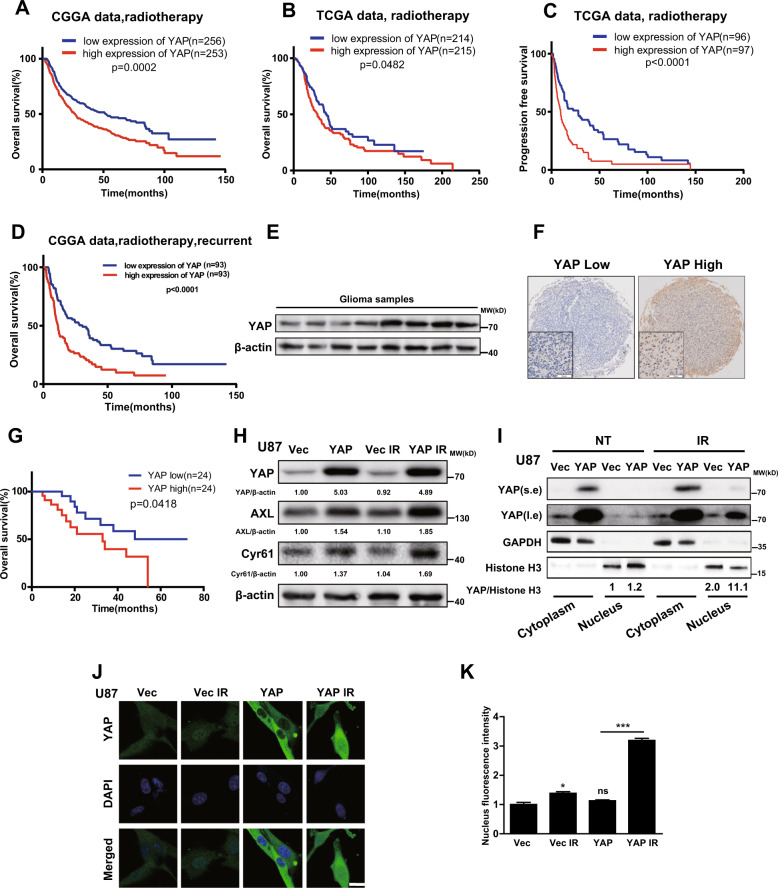
High YAP expression suggests poor prognosis in glioma patients undergoing radiotherapy. **A**, **B** Kaplan–Meier curves showing the overall survival of GBM patients undergoing radiotherapy with different expression levels of YAP from the CGGA and TCGA databases. **C** Kaplan–Meier curves showing the progression-free survival of GBM patients undergoing radiotherapy with different expression levels of YAP from the TCGA database. **D** Kaplan–Meier curves showing the overall survival of recurrent glioma patients accepting radiotherapy with different level of YAP from CGGA database. **E** Representative immunoblots using indicated antibodies in fresh GBM clinical samples to detect the level of YAP (*n* = 27). **F** Representative images of IHC staining of YAP in paraffin-embedded GBM samples (*n* = 21). Scale bars: 50 μm. **G** Kaplan–Meier analysis in GBM patients accepting the radiotherapy after surgical resection with high/low level of YAP. **H** Representative immunoblots using indicated antibodies in U87 cells after radiation (10 Gy). **I** The subcellular level of YAP was detected using cellular fractionation after radiation (10 Gy). Histone and GAPDH were used as the nucleus and cytoplasm loading controls, respectively. Numbers below the bands were the normalized values. **J**, **K** Representative immunofluorescence images of U87 cells stained with anti-YAP (**J**) and quantitative analysis (**K**) of the subcellular location of YAP after radiation (10 Gy).

To study the potential involvement of YAP in radioresistance of gliomas, we first analyzed the CGGA and TCGA databases, and found that in the patients with radiotherapy, high expression of YAP was associated with short overall survival and progression-free survival (Fig. 1A–C). Meanwhile, in recurrent glioma patients accepting radiotherapy, high YAP expression is associated with poor prognosis (Fig. 1D). In addition, we obtained glioma samples during surgical resection and detected the protein levels of YAP in clinical samples using western blotting (Fig. 1E) and TMA combined with IHC assay (Fig. 1F), respectively. We found that patients with high YAP expression had a worse prognosis according to our follow-up results (Fig. 1G). These findings showed that high YAP expression suggests poor prognosis for glioma patients with radiotherapy.

To determine whether radiotherapy affects YAP expression in gliomas, we radiated the glioma cells and found that radiation did not influence the protein level of YAP but increased the protein level of *AXL* and *Cyr61*, two target genes of YAP (Fig. 1H). Importantly, examined by cellular fractionation (Fig. 1I) and immunofluorescence (Fig. 1J, K), the nuclear YAP level increased after radiation. Together, the above results demonstrated that YAP was translocated into the nucleus and activated after radiation.

### YAP exerts radioresistant effects on gliomas

**Fig. 2:**
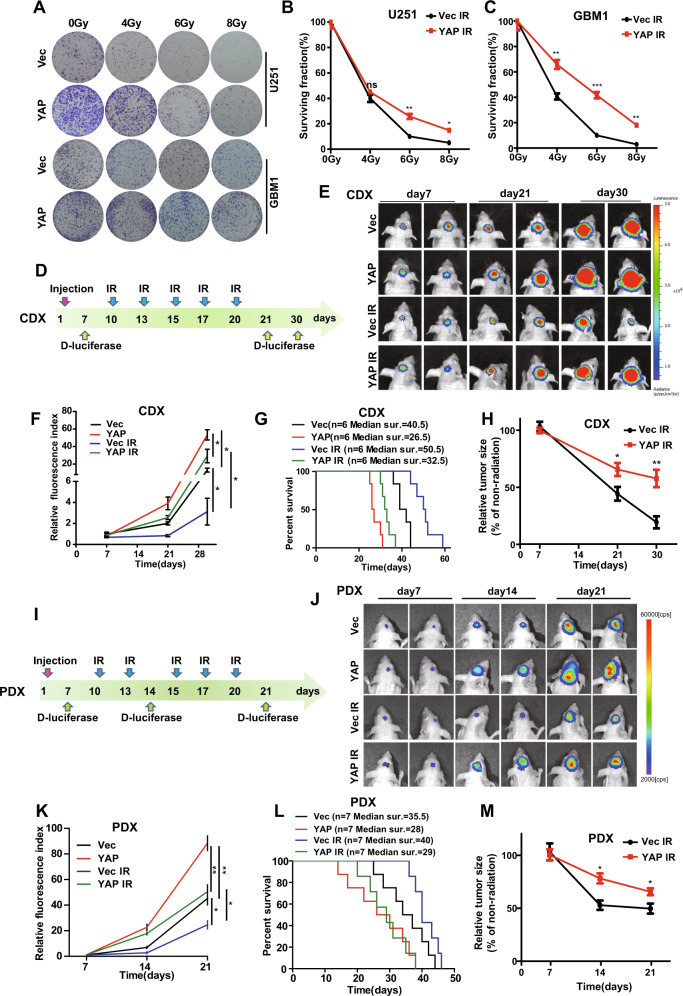
The radioresistant effect of YAP in gliomas. **A**–**C** Representative images (**A**) and quantitative results (**B**, **C**) of colonies of YAP overexpression U251 and GBM1 cells after radiation. **D** Schematic representation of the CDX experimental workflow. **E** Representative pseudocolor bioluminescence images of intracranial xenografts bearing YAP overexpression or vector cells on the indicated days. **F** Quantitative analysis of the tumor size. **G** Kaplan–Meier analysis of the median survival time of mice (*n* = 6). **H** Relative tumor size, which was normalized to % of non-radiation control. **I** Schematic representation of the PDX experimental workflow. **J** Representative pseudocolor bioluminescence images of intracranial xenografts bearing YAP overexpression or vector cells on the indicated days. **K** Quantitative analysis of the tumor size. **L** Kaplan–Meier analysis of the median survival time of mice (*n* = 7). **M** Relative tumor size, which was normalized to % of non-radiation control. CDX: cell-derived xenograft, GBM: primary glioblastoma cells, PDX: patient-derived xenograft, Vec: Vector.

To study the potential role of YAP in glioma radioresistance, we performed a colony formation assay in U251 and primary GBM1 cells. We found that YAP overexpression cells formed more colonies after radiation (Fig. 2A–C). Even glioma cells were treated with low-dose fractionated radiotherapy and YAP overexpression cells also showed higher surviving rates after radiotherapy (sFig. 1A, B). In addition, we performed colony formation assay in the primary GBM1 and GBM2 cells with different endogenous levels of YAP (sFig. 1C). Strikingly, GBM2 cell, which has higher YAP level than GBM1 cell, exhibited poorer response to radiotherapy than GBM1 cells (sFig. 1D, E).

Next, we extended the above experiments in cell-derived xenograft (CDX) and patient-derived xenograft (PDX) models in vivo. As shown in Fig. 2D–F, I–K, mice engrafted with YAP overexpression cells had an increased tumor burden (Fig. 2E, F, J, K) and shorter overall survival time (Fig. 2G, L) after 10 Gy radiation. Inhibition efficiency of radiotherapy was poor in tumors derived from YAP overexpression cells (Fig. 2H, M). Hematoxylin and eosin (HE) staining showed that tumors derived from YAP overexpression cells were significantly larger than those from vector cells after radiation (sFig. 1F, G). The above results indicate that YAP exerts a radioresistant effect on gliomas.

### YAP promotes DNA damage repair of glioma cells after radiation

**Fig. 3:**
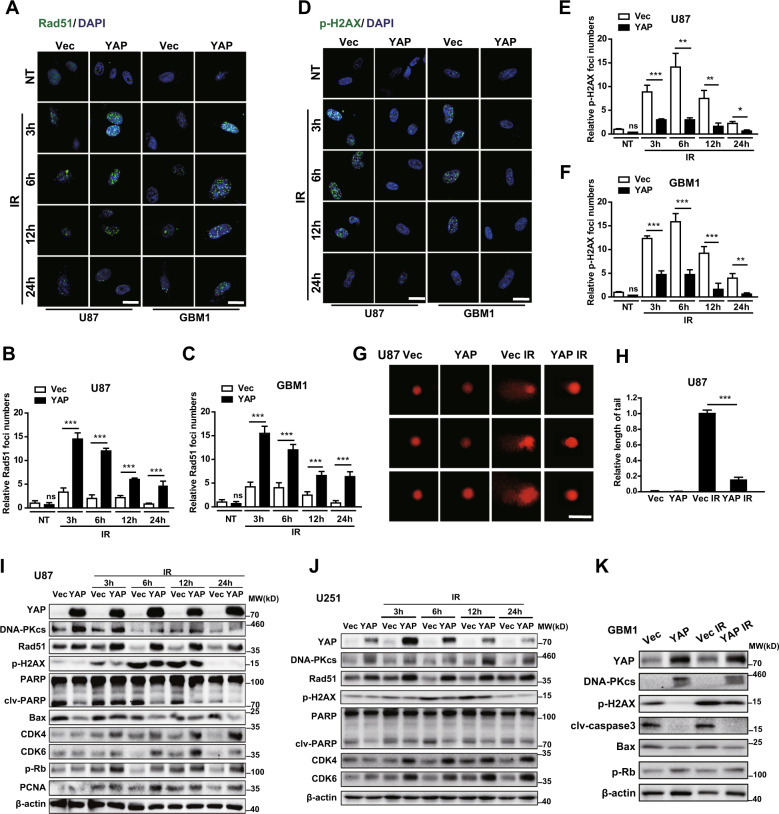
YAP promotes DNA damage repair in glioma cells after radiation. **A**–**C** Representative images of U87 and GBM1 cells stained with anti-Rad51 (**A**) and quantitative analysis (**B**, **C**) after radiation (6 Gy) at the indicated times. **D**–**F** Representative images of U87 and GBM1 cells stained with anti-p-H2AX (**D**) and quantitative analysis (**E**, **F**) after radiation (6 Gy) at the indicated times. **G** Representative images of the comet assay in U87 cells at 6 h after radiation (6 Gy). **H** Quantitative analysis of the length of the comets’ tails. **I**–**K** Representative immunoblots using indicated antibodies in U87 (**I**), U251 (**J**), and GBM1 (**K**) cells after radiation (10 Gy) at the indicated times.

The principal biological effect of radiotherapy is to rapidly erase proliferating cells by inducing DNA damage beyond the cellular capacity to repair [18]. Once DNA was damaged, DNA repair proteins, such as Rad51, are recruited to DNA damage sites to form foci and repair damaged DNA [19]. We found that YAP overexpression cells displayed more numbers of Rad51 foci than vector cells after radiation, not only in U87 but also in GBM cells (Fig. 3A–C). On the contrary, YAP overexpression cells showed less numbers of p-H2AX foci, a marker of DNA damage, both in U87 and GBM1 cells after radiation (Fig. 3D–F). The comet assay showed less DNA damage in YAP overexpression cells at 6 h after radiation (Fig. 3G, H). In addition, overexpression of YAP significantly sped up DNA repair (using DNA–PKcs and Rad51 levels as indicators) and the cell cycle (using CDK4, CDK6, p-RB, and PCNA levels as indicators), while a reduction in DNA damage (using the p-H2AX level as an indicator) and apoptosis (using cleaved PARP and Bax levels as indicators) was observed in U87 (Fig. 3I) and U251 glioma cells (Fig. 3J). Furthermore, upregulation of YAP in primary cultured GBM1 cells significantly accelerated DNA repair and the cell cycle, whereas it inhibited DNA damage and apoptosis after radiation (Fig. 3K), similar to the results obtained from immortalized glioma cells. Consistently, after synchronization, YAP overexpression cells went through the cell cycle faster than the vector cells (sFig. 2A, B). Moreover, GBM2 cells with high endogenous level of YAP significantly accelerated DNA repair and the cell cycle, whereas it inhibited apoptosis after radiation (sFig. 2C). In sharp contrast, cells with YAP downregulation (sFig. 2D) or verteporfin (VP, a blocker of YAP-TEAD interaction) treatment (sFig. 2E) inhibited DNA repair and cell proliferation but promoted cell apoptosis after radiation.

### *FGF2* is a novel target gene of YAP

**Fig. 4:**
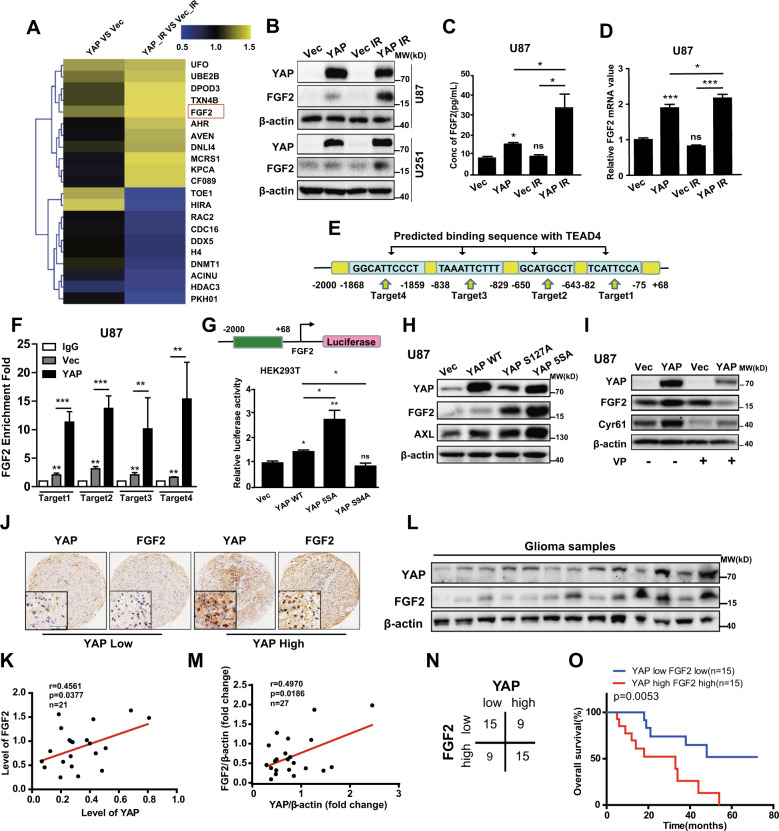
*FGF2* is a novel target gene of YAP. **A** iTraq analysis indicated that the FGF2 protein level increased after YAP overexpression and even more obviously increased after radiation (10 Gy). **B**, **C** The FGF2 protein level was significantly upregulated in YAP overexpression cells after radiation (10 Gy), as shown by western blotting (**B**) and ELISA (**C**). **D** The *FGF2* mRNA level was significantly upregulated in YAP overexpression cells after radiation (10 Gy). **E** Diagram of TEAD4 binding to the *FGF2* promoter and target primer design. **F** ChIP-qPCR analysis of TEAD4 enrichment at the *FGF2* promoter in U87 glioma cells. **G** Diagram of the luciferase reporter driven by the *FGF2* promoter fragment (−2000 to +68 bp). HEK293T cells were transiently transfected with indicated plasmids along with the luciferase reporter. Luciferase activity was detected at 48 h after transfection. **H** Representative immunoblots using indicated antibodies in YAP WT, YAP S127A, and YAP 5SA overexpression U87 cells. **I** Representative immunoblots using indicated antibodies in U87 cells with or without VP (5 mg/L) treatment. **J**, **K** Representative images (**J**) of IHC staining of YAP and FGF2 in paraffin-embedded GBM samples and correlation analysis (**K**) between YAP and FGF2 (*n* = 21). Scale bars: 50 μm. **L**, **M** Representative immunoblots (**L**) using indicated antibodies in fresh GBM clinical samples and correlation analysis (**M**) between YAP and FGF2 (*n* = 27). **N** Correlations of high and low YAP expression with the level of FGF2 (*n* = 48). **O** Kaplan–Meier analysis in GBM patients undergoing radiotherapy with both high/low expression of YAP and FGF2.

Motivated by the above results, we next examined the mechanism through which YAP protects glioma cells from radiation-induced death and promotes DNA repair. By iTraq analysis, we identified the differentially expressed proteins in YAP overexpression cells after radiation and screened out proteins related to DNA repair, the cell cycle, and apoptosis (Fig. 4A). FGF2 was one of the proteins most highly upregulated in YAP overexpression cells, under both non-radiation and radiation conditions (Fig. 4A). Interestingly, FGF2–FGFR signaling has been reported to regulate the response of cells to radiation [20], suggesting that FGF2 may mediate the effects of YAP on radioresistance. Western blotting and enzyme-linked immunosorbent assay (ELISA) showed that FGF2 expression and secretion increased in YAP overexpression cells with or without radiation (Fig. 4B, C). In addition, in YAP overexpression cells, the FGF2 increase was higher after radiation than that without radiation. Furthermore, the mRNA level of FGF2 showed similar results both in U87 and U251 glioma cells (Fig. 4D and sFig. 3A), indicating that *FGF2* may be a target gene of YAP. Next, we used JASPAR (http://jaspar.genereg.net/) to perform transcription factor-binding analysis and predicted five binding sites of TEAD4 in the *FGF2* promoter (Fig. 4E). According to these predicted binding sites, we designed four primer pairs and performed ChIP-qPCR analysis. We found that endogenous TEAD4 could bind to the promoter of *FGF2* and YAP overexpression enhanced the interaction (Fig. 4F). To further analyze whether YAP regulates FGF2 expression at the transcriptional level, a luciferase reporter driven by the *FGF2* promoter fragment (−2000 to +68 bp) (Fig. 4G, upper panel) was co-transfected into HEK293 cells with YAP wild type (WT), YAP 5SA (a constitutive active form of YAP with a serine-to-alanine mutation of all five Hippo pathway target sites), YAP S94A (with a serine-to-alanine mutation at residue 94, which is unable to bind to TEAD), or empty vector as a control. The luciferase reporter assay showed that YAP WT and YAP 5SA enhanced *FGF2* promoter activity, whereas YAP S94A did not (Fig. 4G). In addition, YAP S127A (a constitutive active form of YAP with a serine-to-alanine mutation at residue 127) and YAP 5SA enhanced the FGF2 protein level in U87 cells (Fig. 4H), and similar results were obtained from U251 cells (sFig. 3B). Consistently, the protein levels of FGF2 and Cyr61 decreased after VP treatment (Fig. 4I and sFig. 3C). GBM2 cells with high endogenous level of YAP also had high protein level of FGF2 (sFig. 2C). Therefore, our results indicate that *FGF2* is a novel target gene of YAP.

In line with the above results, the level of YAP was positively correlated with FGF2 both in the CGGA and the TCGA database (sFig. 3D, E). In addition, as shown in Fig. 4J–N, we found that YAP were positively correlated with FGF2 both in paraffin-embedded (Fig. 4J, K) and fresh glioma samples (Fig. 4L, M). More importantly, according to our follow-up results, patients with high YAP and FGF2 expression had a worse prognosis than those with low YAP and FGF2 expression (Fig. 4O).

### FGF2 mediates the radioresistant effect of YAP on gliomas

**Fig. 5:**
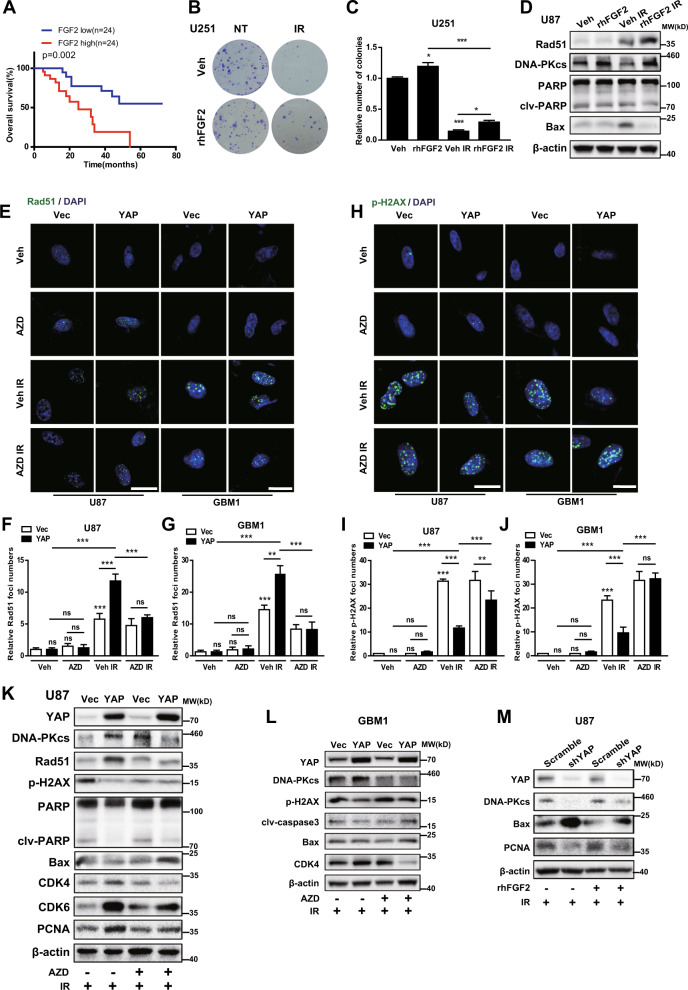
FGF2 mediates the radioresistant effect of YAP on gliomas. **A** Kaplan–Meier analysis in GBM patients undergoing radiotherapy with high/low expression levels of FGF2 (*n* = 48). **B**, **C** Representative images (**B**) and quantitative results (**C**) of colonies in U251 cells treated with rhFGF2 and/or radiation (IR: 6 Gy; rhFGF2: 25 ng/mL). **D** Representative immunoblots using indicated antibodies in U87 cells with different treatments (IR: 10 Gy; rhFGF2: 25 ng/mL). **E**–**G** Representative images of U87 and GBM1 cells stained with anti-Rad51 (**E**) and quantitative results (**F**, **G**) after different treatments (IR: 6 Gy; AZD4547: 1 μM). **H**–**J** Representative images of U87 and GBM1 cells stained with anti-p-H2AX (**H**) and quantitative results (**I**, **J**) after different treatments (IR: 6 Gy; AZD4547: 1 μM). **K**–**M** Representative immunoblots using indicated antibodies in glioma cells with YAP overexpression (**K**, **L**) or downregulation (**M**) and different treatments (IR: 10 Gy; AZD4547: 1 μM; rhFGF2: 25 ng/mL). AZD: AZD4547, Veh: Vehicle.

According to our follow-up results, patients undergoing radiotherapy with high FGF2 expression had a worse prognosis than those with low FGF2 expression (Fig. 5A). Consistently, we found that recombinant human FGF2 (rhFGF2) treatment promoted the proliferation (Fig. 5B, C) and DNA repair of glioma cells but inhibited apoptosis after radiation (Fig. 5D), similar to the effects of YAP. We therefore wonder whether FGF2 mediates the radioresistant effect of YAP on gliomas. As a member of the FGF family, FGF2 plays an important role in cell growth and differentiation, which is dependent on FGF2–FGFR signaling [21]. We then used AZD4547, an inhibitor of FGFR, to block the effect of FGF2. As shown in Fig. 5E–G, AZD4547 has no effect on Rad51 foci in vector and YAP overexpression cells without radiation. However, AZD4547 treatment partially abolished the stimulatory effect of YAP on Rad51 foci after radiation (Fig. 5E–G). Correspondingly, AZD4547 specifically blocked the inhibitory effect of YAP overexpression on p-H2AX foci after radiation (Fig. 5H–J). AZD4547 also impeded the promotive effect of YAP on the cell cycle post radiation (sFig. 4). Similarly, as shown in Fig. 5K, L, the effects of YAP overexpression on DNA repair, DNA damage, apoptosis, and the cell cycle were attenuated after AZD4547 administration in U87 and GBM1 cells. On the contrary, YAP downregulation inhibited DNA repair and cell proliferation but promoted apoptosis after radiation (Fig. 5M, lanes 1 and 2). Importantly, the above results were rescued to some extent in the presence of rhFGF2 (Fig. 5M). Taken together, these results suggest that FGF2 mediates the radioresistant effect of YAP on glioma cells.

### The MAPK–ERK pathway is required for the radioresistant effect of YAP

It has been reported that FGF2 interacts with FGFR to form a complex and then activates downstream signaling, including phosphatidylinositol 3-kinase (PI3K)–AKT, MAPK–extracellular-signal-regulated kinase (ERK), and Janus kinase-signal transducer and activator of transcription (JAK–STAT) [22]. Notably, examined by iTraq analysis and as shown in sFig. 5A, the MAPK–ERK signaling pathway is one of the most altered pathways in YAP overexpression cells after radiation, indicating that FGF2 may exert its effect through MAPK–ERK signaling. Indeed, among the three FGF2–FGFR downstream pathways, the p-ERK level (the indicator of MAPK–ERK signaling activity) increased most after YAP overexpression and radiation, and AZD4547 treatment abolished this effect both in U87 and in GBM1 cells (sFig. 5B). In addition, the promotive effect of YAP on Rad51 foci (sFig. 6A–C) and the inhibitory effect of YAP on p-H2AX foci were abrogated by U0126, a well-known MAPK–ERK pathway inhibitor (sFig. 6D–F). Consistently, the effects of YAP and FGF2 on the MAPK–ERK pathway (using p-ERK level as the indicator), DNA repair, apoptosis, and the cell cycle after radiation were attenuated after U0126 treatment in U87 and GBM1 glioma cells (sFig. 6G, H). The above evidence indicates that the MAPK–ERK pathway is required for the radioresistant effect of YAP.

### Blocking YAP–FGF2–MAPK sensitizes gliomas to radiotherapy

**Fig. 6:**
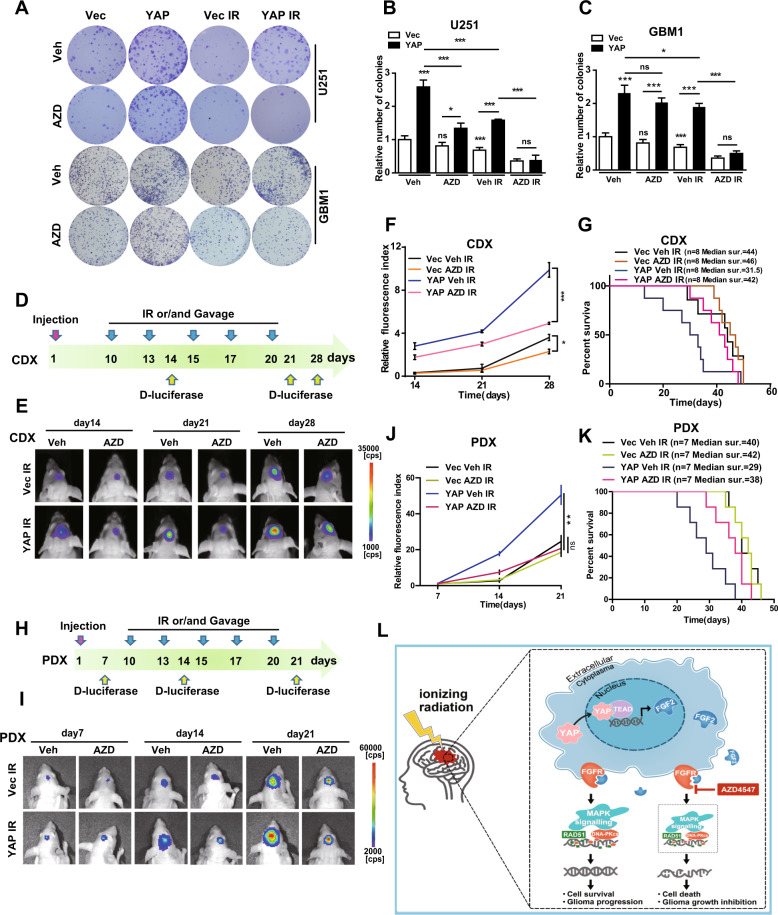
Blocking YAP–FGF2–MAPK sensitizes gliomas to radiotherapy. **A**–**C** Representative images (**A**) and quantitative results (**B**, **C**) of colonies in YAP overexpression U251 and GBM1 cells with indicated treatments (IR: 6 Gy; AZD4547: 1 μM). **D** Schematic representation of the CDX experimental workflow. **E** Representative pseudocolor bioluminescence images of intracranial xenografts bearing YAP overexpression or vector U87 cells with different treatments on the indicated days. **F** Quantitative analysis of the fluorescence index. **G** Kaplan–Meier analysis of the median survival time of mice (*n* = 8). **H** Schematic representation of the PDX experimental workflow. **I** Representative pseudocolor bioluminescence images of intracranial xenografts bearing YAP overexpression or vector GBM1 cells with different treatments on the indicated days. **J** Quantitative analysis of the fluorescence index. **K** Kaplan–Meier analysis of the median survival time of mice (*n* = 7). AZD: AZD4547, Veh: Vehicle. **L** Working model: YAP was translocated from the cytoplasm into the nucleus after radiation, where it promotes the transcription and secretion of FGF2, which activates the MAPK–ERK pathway to promote DNA damage repair and exerts radioresistant effects. Blocking YAP–FGF2–MAPK by AZD4547 sensitizes gliomas to radiotherapy.

To expand upon the significance, we sought to develop a preclinical model to determine whether inhibiting YAP–FGF2–MAPK would sensitize tumors to radiation. The colony formation assay indicated that YAP knockdown cells (sFig. 7A, B) and cells treated with VP (sFig. 7C, D) showed stronger responses to radiotherapy. The promotive effect of YAP on cell proliferation after radiation was blocked by AZD4547 (Fig. 6A–C) and U0126 (sFig. 7E–H) in immortalized and GBM1 cells. Importantly, as AZD4547 is able to penetrate the blood–brain barrier [5], mice orthotopically engrafted with immortalized U87 (Fig. 6D) and primary GBM1 (Fig. 6H) cells were orally administered with AZD4547. Notably, AZD4547 treatment blocked the radioresistant effect of YAP, both in CDX (Fig. 6D–F) and PDX (Fig. 6H–J) models. After AZD4547 administration and radiation treatment, mice with YAP overexpression tumors exhibited a significant inhibition of tumor growth (Fig. 6E, F, I, J) and a corresponding increase in survival (Fig. 6G, K). These results indicate that blocking YAP–FGF2–MAPK sensitizes gliomas to radiotherapy.

## Discussion

### YAP was activated after radiation and exerts radioresistant effects on gliomas

Radiotherapy, which causes DNA double-strand breaks, leading to cell cycle exit and apoptosis, is considered as the first treatment choice for glioma. However, despite great technological improvements, the effect of radiotherapy is generally limited because of the marked radioresistance of glioma cells. Previously, we demonstrated that YAP promotes glioma malignant progression and is a potential therapeutic target in gliomas [13, 14]. In this study, we found that YAP is translocated from the cytoplasm into the nucleus after radiation, where it promotes the transcription and secretion of FGF2, which activates the MAPK–ERK pathway and promotes DNA damage repair, leading to radioresistance. Blocking YAP–FGF2–MAPK sensitizes gliomas to radiotherapy (Fig. 6L).

In previous studies, YAP activity abrogates cell cycle checkpoints, enables cells to enter mitosis with unrepaired DNA, and accelerates tumor growth and ongoing proliferation after radiation in shh-driven medulloblastoma [17]. However, in our study, YAP stimulates glioma cell proliferation and inhibits apoptosis after radiation via accelerating DNA repair and the cell cycle. Interestingly, Zhang et al. [23] reported that inhibition of the transcriptional co-activator with PDZ-binding motif (TAZ), but not the paralog of YAP, contributes to radiation-induced senescence and growth arrest in glioma cells. It has also been reported that radiation affects the level of TAZ but not YAP [23]. Although the level of YAP also showed no change in our study, we found that YAP was translocated into the nucleus after radiation. From a clinical perspective, our findings reveal that glioma patients with high YAP expression had shorter recurrent times and worse prognosis after radiation. Taken together, we demonstrated that YAP is implicated in the promotion of cell survival and growth in response to radiotherapy in gliomas.

### *FGF2* is a novel target gene of YAP and FGF2–MAPK pathway mediates the effects of YAP on radioresistance

Taking advantage of iTraq assay, we found that, after radiation, the growth factor FGF2 is one of the most strongly upregulated proteins in YAP overexpression cells. After radiation, YAP was translocated into the nucleus, where it promotes the transcription and secretion of FGF2, which speeds up the proliferation of glioma cells by enhancing the DNA damage repair, similar to the effects of YAP. Previously, Han et al. [24] reported that YAP enhances FGF-dependent neural stem cell proliferation by inducting FGFR expression independently of TEAD. In addition, YAP overexpression increased the mRNA and protein levels of FGF2 [25], whereas YAP downregulation decreased the protein level of FGF2 [26]. Unfortunately, both studies just examined the mRNA and protein levels of FGF2 after YAP manipulation and did not check whether *FGF2* is a target gene of YAP. Based on our knowledge, combining western blotting and ChIP-qPCR with luciferase assays, our data identified, for the first time, that *FGF2* is a novel target gene of YAP.

FGF signaling plays an important role in the pathogenesis of diverse tumor types and clinical reagents that specifically target FGFs or FGFRs are being developed [27, 28]. It has been reported that high-molecular-weight forms of FGF2 play a radioprotective role in ovarian cancer cells [29, 30]. Secretion of FGF2 by glioblastoma cells enhances the blood–brain barrier function of endothelial cells, contributing to drug resistance [31]. FGFR1 induces glioblastoma radioresistance through the PLCγ and HIF-1α pathways, and inhibition of FGFR1 radiosensitizes glioblastoma cells [32]. Inhibition of FGF using the small-molecule multi-FGF receptor blocker SSR128129 radiosensitizes human glioblastoma [20], indicating that targeting of the FGF2–FGFR pathway might be of interest when aiming to radiosensitize human glioblastoma. FGF2–FGFR is mainly involved in three pathways, including MAPK–ERK, PI3K–AKT, and JAK–STAT3. As a classical signaling pathway involved in tumor development and cancer progression, a series of studies have demonstrated that the MAPK–ERK pathway plays a role in glioma radioresistance. When the cells were treated with a MAPK inhibitor, non-homologous end joining was blocked [4]. U0126 treatment and ERK silencing reduced the levels of DNA repair proteins in glioma cells after radiation [33]. In this study, the promotive effects of YAP on DNA repair and radioresistance were blocked by AZD4547 and U0126, suggesting that the FGF2–MAPK pathway mediates the effects of YAP on radioresistance.

### YAP–FGF2–MAPK is an actionable target for improving radiotherapy efficacy

The above results shed light on targeting YAP–FGF2–MAPK as a novel therapeutic approach to restore radiosensitivity of gliomas. In our study, downregulation YAP or inhibition of YAP by VP could radiosensitize human gliomas. The promotive effects of YAP–FGF2 on radioresistance were blocked by U0126. Blocking FGF2 signaling with the FGFR inhibitor AZD4547, which is evaluated in clinical trials as anticancer drugs [34], abolished YAP-conferred radioresistance and subsequent malignant progression of gliomas both in vitro and in vivo. Neither VP nor U0126 could penetrate the blood–brain barrier, but AZD4547 could. This suggests that AZD4547 may become a clinical drug to overcome the resistance of radiotherapy in the future.

In conclusion, high YAP expression suggests poor prognosis in glioma patients undergoing radiotherapy. Radiation activates YAP contributing to glioma cell growth via driving the expression of FGF2 and subsequently activating the MAPK–ERK pathway. We identified that *FGF2* is a new target gene of YAP. Activation of the YAP–FGF2–MAPK pathway endows glioma cells with the ability to enhance DNA repair, promote cell cycle progression, and inhibit apoptosis, leading to cell survival after radiation. Collectively, our novel findings clarify an association between oncogenic YAP and radioresistance, suggesting that targeting of the YAP–FGF2–MAPK pathway by AZD4547 may have a therapeutic value for glioma patients by restoring radiosensitivity and inducing glioma cell death after radiation.

## Materials and methods

### Cell culture

The U251 glioma cell was purchased from Shanghai Cell Bank, Chinese Academy of Science. The U87 glioma cell was purchased from American Type Culture Collection. All cells were cultured in Dulbecco’s modified Eagle’s medium (Gibco) supplemented with 10% fetal bovine serum (Gibco). Primary glioblastoma cell culture was performed according to our previous studies [13].

### Antibodies, reagents, and plasmids

See the Supplementary Materials and Methods for details.

### Radiation treatment

For radiation treatment, cells were seeded in a Petri dish or a six-well plate 24 h before the treatment. The mice and cells were exposed to X-rays at indicated times using a Varian 23EX radiator (Varian) with indicated doses.

### Colony formation assay

Colony formation assay was performed according to our previous studies [35, 36].

### Intracranial cell-derived and patient-derived xenograft models, and in vivo imaging analysis in nude mice

All in vivo experiments were approved by the Institutional Ethics Committee and met the standards required by the guidelines of Xuzhou Medical University. The intracranial glioma model was established in nude mice according to our previous study [37], except that the glioma cells were labeled with GFP-luci. See the Supplementary Materials and Methods for further details.

### Immunofluorescence

At the indicated times post radiation, cells were fixed and probed with primary antibodies. The nuclei were labeled with 4′,6-diamidino-2-phenylindole (DAPI), and cells were embedded and photographed by confocal microscopy (Zeiss 710).

### Comet assay

The comet assay was performed with the Trevigen kit according to the manufacturer’s instructions. Briefly, glioma cells were collected at 0 and 6 h after radiation at 10 Gy. The single-cell suspension was mixed with low-melting agarose and coated on a glass slide, which was then immersed in lysis solution. After the cells were lysed, gel electrophoresis and neutralization of slides were performed. Analysis of DNA migration was performed by staining DNA with ethidium bromide and slides were photographed digitally (Nikon Eclipse E800). The tail length was calculated using Comet Assay IV software.

### Flow cytometry

Cell cycle analysis was performed by flow cytometry with a commercial kit (propidium iodide/RNase Staining Buffer, BD) according to the manufacturer’s instructions.

### iTraq labeling and liquid chromatography with tandem mass spectrometry analysis

See the Supplementary Materials and Methods for details.

### Enzyme-linked immunosorbent assay

Supernatant from glioma cells after radiation was collected. FGF2 levels were examined using the R&D ELISA kit according to the manufacturer’s instructions.

### RNA extraction and quantitative PCR

See the Supplementary Materials and Methods for details.

### Chromatin immunoprecipitation

Chromatin immunoprecipitation (ChIP) assays were performed using a Pierce Magnetic ChIP kit (Thermo Scientific) according to the manufacturer’s instructions. Briefly, cells were crosslinked, lysed, and sonicated to generate DNA fragments. ChIP was performed using antibodies against TEAD4 or normal IgG as a control. The immunoprecipitates were then washed and eluted. The eluates were de-crosslinked and ChIP-enriched DNA was purified for quantitative PCR (qPCR) analysis using the following primers: target 1: Forward: 5′-TACAAAAAATTAGCCCTGCGTG-3′, Reverse: 5′-AATGGCACGATCTCGGCT-3′;

target 2: Forward: 5′-CCTTCATTCCAGAGGTGCCTT-3′, Reverse: 5′-GGGACTTACTTAGCAAGGCCTATT-3′;

target 3: Forward: 5′-GGCAACAAGAGCAAAACTCTGT-3′, Reverse: 5′-CAGTAGAAAGTAGGCCATAGAAGAGTA-3′;

target 4: Forward: 5′-GTGGAGCCCAGGGAATGC-3′, Reverse: 5′-TCCGCTAATCTGGCACCC-3′.

### Cell luciferase assay

The luciferase reporter plasmid was constructed by cloning the *FGF2* promoter into the pGL4.10-Basic vector. For the luciferase reporter assay, HEK293T cells were transfected with the luciferase reporter and indicated plasmids. At 48 h after transfection, cells were lysed and luciferase activity was assayed using the Dual-Luciferase Reporter Assay System (Promega) following the manufacturer’s instructions.

### Cellular fractionation and western blotting

Cellular fractionation was conducted by using Membrane and Cytosol Protein Extraction Kit (Beyotime). Western blotting was performed according to previous study [38].

### Glioma samples and tissue microarray

Glioma samples including 21 paraffin-embedded tissues and 27 fresh samples were collected from the Affiliated Hospital of Xuzhou Medical University. The tissue microarray (TMA) was constructed as described previously [39]. Fresh samples were collected immediately after surgical resection and stored at −80 °C. Surgically removed tissues were sampled for histological diagnosis according to the World Health Organization classification. No patient has received chemotherapy, immunotherapy, or radiotherapy before surgical resection. After surgical resection, all these patients were treated with radiotherapy. Written informed consent was obtained from the patients and the study was approved by the institutional Ethics Committee. Clinical histories were recorded at screening.

### Immunohistochemistry

The immunohistochemistry (IHC) was performed as described previously [39]. See the Supplementary Materials and Methods for details.

### Kaplan–Meier analysis of the online database

Survival analysis of data on glioma patients downloaded from the Chinese Glioma Genome Atlas (CGGA) database (http://www.cgga.org.cn) and The Cancer Genome Atlas (TCGA) database (https://portal.gdc.cancer.gov/) was performed using Kaplan–Meier analysis. The cutoff values for separating high- and low-expression groups of YAP or FGF2 were determined by an auto scan. The Kaplan scanner was used to determine the best cutoff values for the level of gene expression.

### Statistical analysis

The results are representative of experiments repeated at least three times and presented as mean ± SEM. Statistical comparisons of data from the experiments on cultured cells or mice were performed using the two-tailed Student’s *t*-test or one-way analysis of variance for multiple comparisons followed by Dunnett’s *t*-test for post hoc pairwise comparisons. Overall survival curves were drawn using the Kaplan–Meier method and compared using the log-rank test. *P*-values < 0.05 were considered to indicate statistical significance (*P < 0.05, **P < 0.01, ***P < 0.001).

## Supplementary information

Supplementary Materials and Methods

Supplementary Figure 1

Supplementary Figure 2

Supplementary Figure 3

Supplementary Figure 4

Supplementary Figure 5

Supplementary Figure 6

Supplementary Figure 7
