# Supplementary material for: Radiation-induced YAP activation confers glioma radioresistance via promoting FGF2 transcription and DNA damage repair
Source: Oncogene. 2021 Jun 14;40(27):4580–91. doi: 10.1038/s41388-021-01878-3 (PMC8266683; doi:10.1038/s41388-021-01878-3)
Supplement: Supplementary file 1 — Supplementary Materials and Methods [file 41388_2021_1878_MOESM1_ESM.docx]

**Supplementary Materials and Methods**

**Antibodies, reagents and plasmids**

Rabbit monoclonal anti-YAP, mouse monoclonal anti-β-actin, rabbit polyclonal anti-Rad51, and rabbit monoclonal anti-PCNA, rabbit polyclonal anti-Histone-H3, mouse monoclonal anti-TEAD4 were from Abcam. Mouse monoclonal anti-GAPDH, rabbit monoclonal anti-p-H2AX, rabbit monoclonal anti-DNA-PKcs, rabbit monoclonal anti-PARP, rabbit monoclonal anti-PARP, rabbit monoclonal anti-CDK4, mouse monoclonal anti-CDK6, rabbit monoclonal anti-p-Rb, rabbit monoclonal anti-FGF2, rabbit monoclonal anti-ERK, rabbit monoclonal anti-p-ERK, rabbit monoclonal anti-p-Rb, mouse monoclonal anti-STAT3, rabbit monoclonal anti-p-STAT3, rabbit monoclonal anti-AKT, rabbit monoclonal anti-p-AKT, and rabbit monoclonal anti-cleaved-caspase3 were from Cell signaling. Rabbit monoclonal anti-Bax was from Santa Cruz. AZD4547 and U0126 were purchased from TargetMol. Recombinant human FGF2 (rhFGF2) was bought from Peprotech. Verteporfin (VP) was bought from Sigma.

Control and YAP wild type (WT) plasmids were kindly gifted by Prof. Hongbin Ji at the Institute of Biochemistry and Cell Biology, Shanghai Institutes for Biological Sciences, Chinese Academy of Sciences[[1](#_ENREF_1)]. YAP S94A, YAP S127A and YAP 5SA plasmids were kindly gifted by Prof. Bin Zhao at the Life Sciences Institute of Zhejiang University[[2](#_ENREF_2)]. pGL4.10-FGF2 promoter plasmid was bought from OBiO (Shanghai, China). pLL3.7- YAP (shYAP) was constructed in our previous reports[[3](#_ENREF_3), [4](#_ENREF_4)].

**Intracranial cell-derived and patient-derived xenograft models and *in vivo* imaging analysis in nude mice**

Female athymic BALB/c nude mice (4 weeks, 20 g) were purchased from Charles River (Beijing, China) and GemPharmatech (Nanjing, China). The intracranial glioma model was established in nude mice according to our previous study [[5](#_ENREF_5)]. Briefly, 1 × 10^6^ U87 cells or primary glioblastoma cells (GBM) with GFP-luci in 5 μL Leibovitz’s L-15 medium (Gibco, USA) were injected into the right striatum of mice (*n* ≥ 6 per group). The mice were exposed to X-rays at day 10, 13, 15, 17, and 20 (2 Gy per time, totally 10 Gy) after transplantation. For some experiments, the mice were treated with AZD4547 (50 mg/kg) by gavage 4 h before radiation. The intensity of luciferase fluorescence of the nude mice was observed under a Xenogen IVIS Spectrum optical imaging device (Caliper, USA) at the designated days after transplantation.

To draw the survival curve, the mice were sacrificed when they exhibited hemiplegia, listlessness, cachexia, and other neurological symptoms. The brain cryosections were subjected to HE staining, and the tumor volume was calculated according to the formula V = 0.5 × ab^2^, with “a” representing the longest diameter, and “b” representing the shortest diameter.

**RNA extraction and quantitative PCR**

Total RNA of glioma cells was extracted and reversely transcribed into cDNA. The cDNA was amplified using SuperReal PreMix Plus (SYBR Green, Tiangen), and amplifications were carried out using the Applied Bio-systems 7500. The products of cDNA were amplified using SuperReal PreMix Plus (SYBR Green, Tiangen), and amplifications were carried out using the Applied Bio-systems 7500. Primer sequences for quantitative PCR were as follows:

YAP-F: 5′-CACAGCTCAGCATCTTCGAC-3′,

YAP-R: 5′-TATTCTGCTGCACTGGTGGA-3′,

FGF2-F: 5′-TGGCAGCCGGGAGCATCAC-3′,

FGF2-R: 5′-TTTGCAGTACAGCCGCTTGGG-3′,

β-actin-F: 5′-CCAACCGCGAGAAGATGA-3′,

β-actin-R: 5′-CCAGAGGCGTACAGGGATAG-3′.

**iTraq labeling and LC-MS/MS analysis**

Cell lysates were labeled with isobaric tags for relative and absolute quantification (iTraq) reagents. The labeled peptides were purified and separated by liquid chromatography (LC). The iTraq-labeled peptide mixtures were reconstituted and eluted. The peptides were eluted onto a C18 column packed in-house, and the data were acquired. The above experiments were conducted by BGI (China).

**Immunohistochemistry**

Briefly, paraffin sections were heated at 60°C, deparaffinized in xylene, rehydrated in graded ethanol, and microwaved for antigen retrieval. The sections were treated with 3% hydrogen peroxide for 30 min. Slides were incubated at 4°C overnight with primary antibodies. The bound antibodies were detected by use of a streptavidin–peroxidase kit (Zhongshan Gold Bridge Bio, Beijing, China). The slides were counterstained with hematoxylin, dehydrated with ethanol and xylene, and covered with coverslips. The results were presented as the percentage of the glioma cells with positive staining.

**References**

1. Huang H, Zhang W, Pan Y, Gao Y, Deng L, Li F, et al. YAP Suppresses Lung Squamous Cell Carcinoma Progression via Deregulation of the DNp63-GPX2 Axis and ROS Accumulation. Cancer research. 2017;77:5769-81.

2. Zhang Q, Liu N, Bai J, Zhou Q, Cong Yh. Human telomerase reverse transcriptase is a novel target of Hippo℡AP pathway. Faseb Journal. 2020.

3. Wang Y, Pan P, Wang Z, Zhang Y, Xie P, Geng D, et al. β-catenin-mediated YAP signaling promotes human glioma growth. Journal of Experimental & Clinical Cancer Research. 2017;36:136.

4. Zhang Y, Xie P, Wang X, Pan P, Wang Y, Zhang H, et al. YAP Promotes Migration and Invasion of Human Glioma Cells. J Mol Neurosci. 2018;64:262-72.

5. Liu X, Lu D, Ma P, Liu H, Cao Y, Sang B, et al. Hugl-1 inhibits glioma cell growth in intracranial model. Journal of Neuro-Oncology. 2015;125:113-21.

**Supplementary figure legends**

**sFig. 1 The radioresistant effect of YAP on gliomas**

**A and B.** Representative images (A) and quantitative results (B) of colonies after glioma cells treated with low-dose fractionated radiation (2Gy per time x 2, totally 4Gy) or single high dose radiation (4Gy). **C.** Representative immunoblots using indicated antibodies in GBM1 and GBM2 cells. **D and E.** Representative images (D) and quantitative results (E) of colonies in the primary GBM1 and GBM2 cells with different endogenous levels of YAP after radiotherapy. **F and G.** HE staining (**F**) and quantitative analysis (**G**) of the intracranial xenografts. Scale bar: 1 mm.

**sFig. 2 YAP promotes DNA damage repair in glioma cells after radiation**

**A and B.** Cell cycle profiles were examined in U87 cells after radiation (6 Gy) at the indicated times. **C.** Representative immunoblots using indicated antibodies in the primary GBM1 and GBM2 cells with different endogenous levels of YAP after radiation (10 Gy). **D.** Representative immunoblots using indicated antibodies in YAP-downregulation U87 cells after radiation (10 Gy). **E.** Representative immunoblots using indicated antibodies in U87 cells with VP (5 mg/L) treatment after radiation (10 Gy).

**sFig. 3. FGF2 is a target gene of YAP**

**A.** The *FGF2* mRNA level was significantly upregulated in YAP overexpression U251 cells after radiation (10 Gy). **B.** Representative immunoblots using indicated antibodies in YAP WT, YAP 5SA and YAP S94A overexpression U251 cells. **C.** Representative immunoblots using indicated antibodies in U251 cells with or without VP (5 mg/L) treatment. **D and E.** The correlation between YAP and FGF2 in glioma specimens from CGGA database (n=639) and TCGA database (n=429).

**sFig. 4. FGF2 mediates the radioresistant effect of YAP on glioma cells.**

Cell cycle profiles examined in U87 cells under different treatments (IR: 6 Gy; AZD4547: 1 μM).

**sFig. 5. FGF2–MAPK pathway mediates the effects of YAP on radioresistance**

**A.** KEGG analysis of significantly changed pathways examined by iTraq analysis after YAP over-expression and radiation. **B.** The activation of MAPK signaling was attenuated upon AZD4547 treatment in YAP overexpression U87 and GBM1 cells after radiation (IR: 10 Gy; AZD4547: 1 μM).

**sFig. 6. FGF2–MAPK pathway mediates the effects of YAP on DNA damage repair**

**A–C.** Representative images of U87 and GBM1 cells stained with anti-Rad51 (**A**) and quantitative results (**B and C**) after different treatments (IR: 6 Gy; U0126: 20 μM). **D–F.** Representative images of U87 and GBM1 cells stained with anti-p-H2AX (**D**) and quantitative results (**E and F**) after different treatments (IR: 6 Gy; U0126: 20 μM). **G.** Representative immunoblots using indicated antibodies in U87 cells with YAP overexpression and different treatments (IR: 10 Gy; U0126: 20 μM). **H.** Representative immunoblots using indicated antibodies in GBM1 cells with different treatments (IR: 10 Gy; U0126: 20 μM; rhFGF2: 25 ng/mL).

**sFig. 7. YAP-FGF2-MAPK is an actionable target for improving radiotherapy efficacy**

**A–D.** Representative images (**A and C**) and quantitative results of colonies of YAP knockdown (**B**) or VP-treated (5 mg/L, **D**) U251 glioma cells after radiation. **E–H.** Representative images (**E and G**) and quantitative results (**F and H**) of colonies in YAP overexpression U251 and GBM1 cells with U0126 and radiation treatments (IR: 4 Gy; U0126: 20 μM).
