# Supplementary figures and images for: Radiation-induced YAP activation confers glioma radioresistance via promoting FGF2 transcription and DNA damage repair
Source: Oncogene. 2021 Jun 14;40(27):4580–91. doi: 10.1038/s41388-021-01878-3 (PMC8266683; doi:10.1038/s41388-021-01878-3)

**sFig.1**

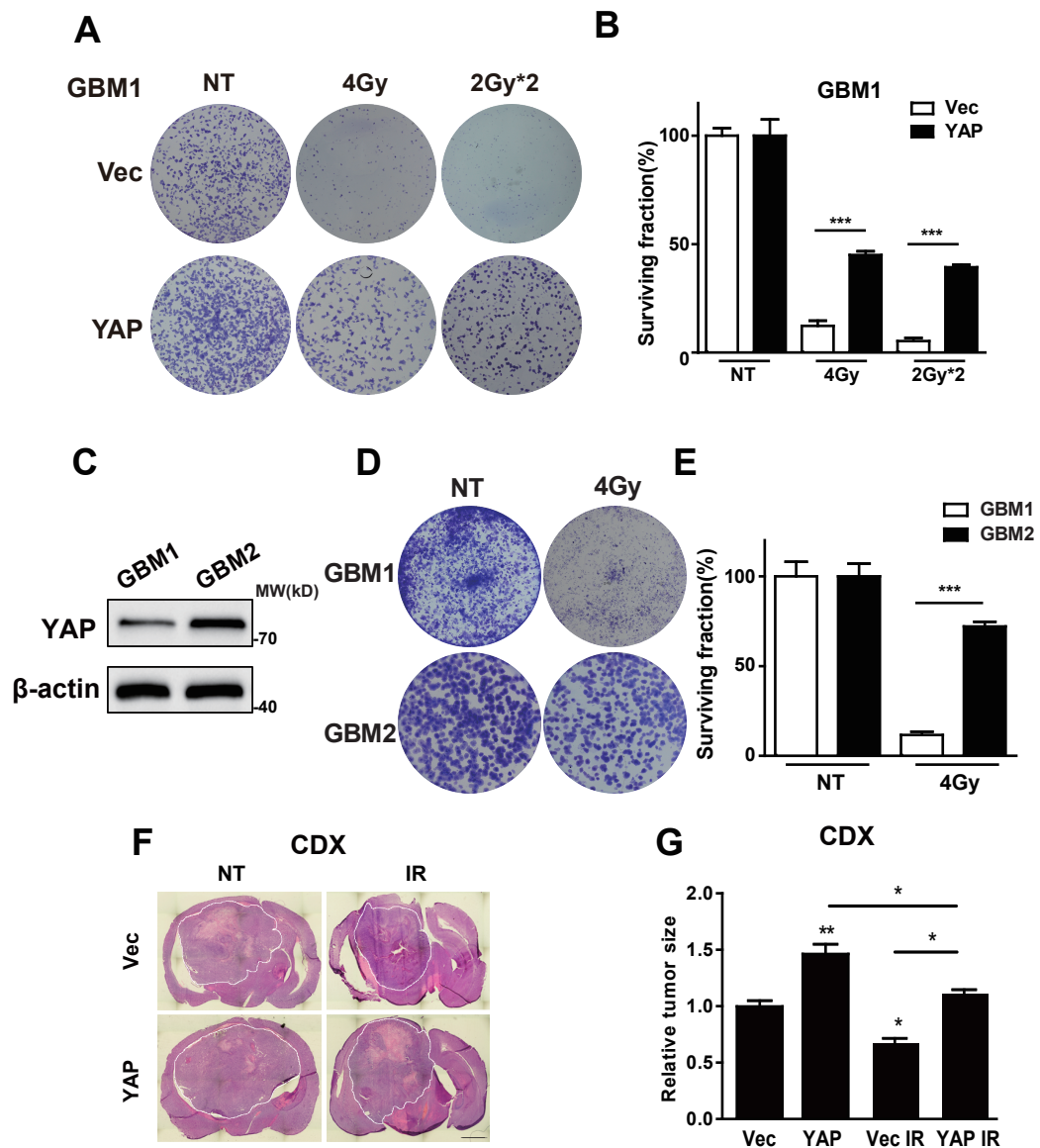

Supplement: Supplementary file 2 — Supplementary Figure 1 [file 41388_2021_1878_MOESM2_ESM.pdf]

**sFig.2**

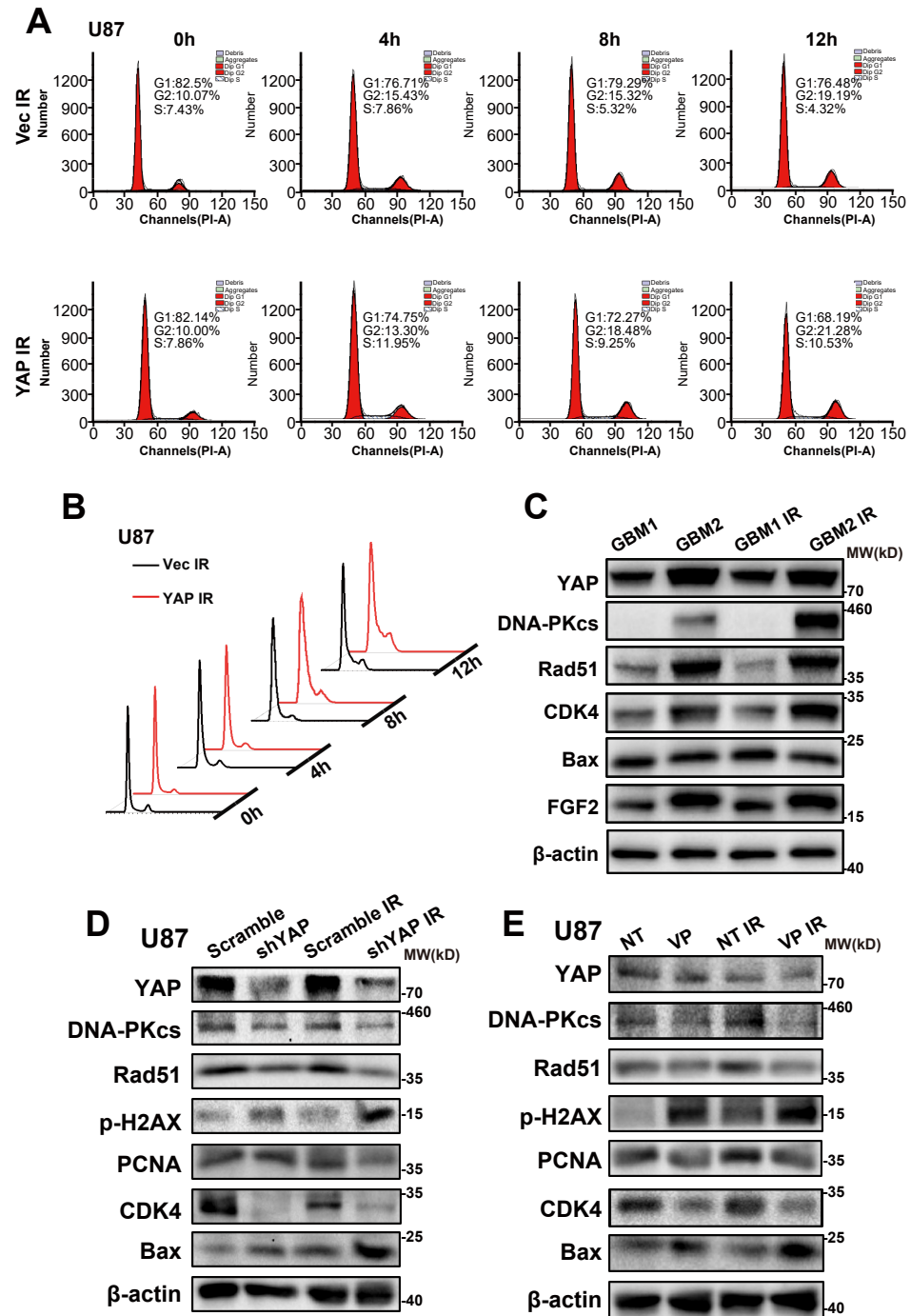

Supplement: Supplementary file 3 — Supplementary Figure 2 [file 41388_2021_1878_MOESM3_ESM.pdf]

sFig.3

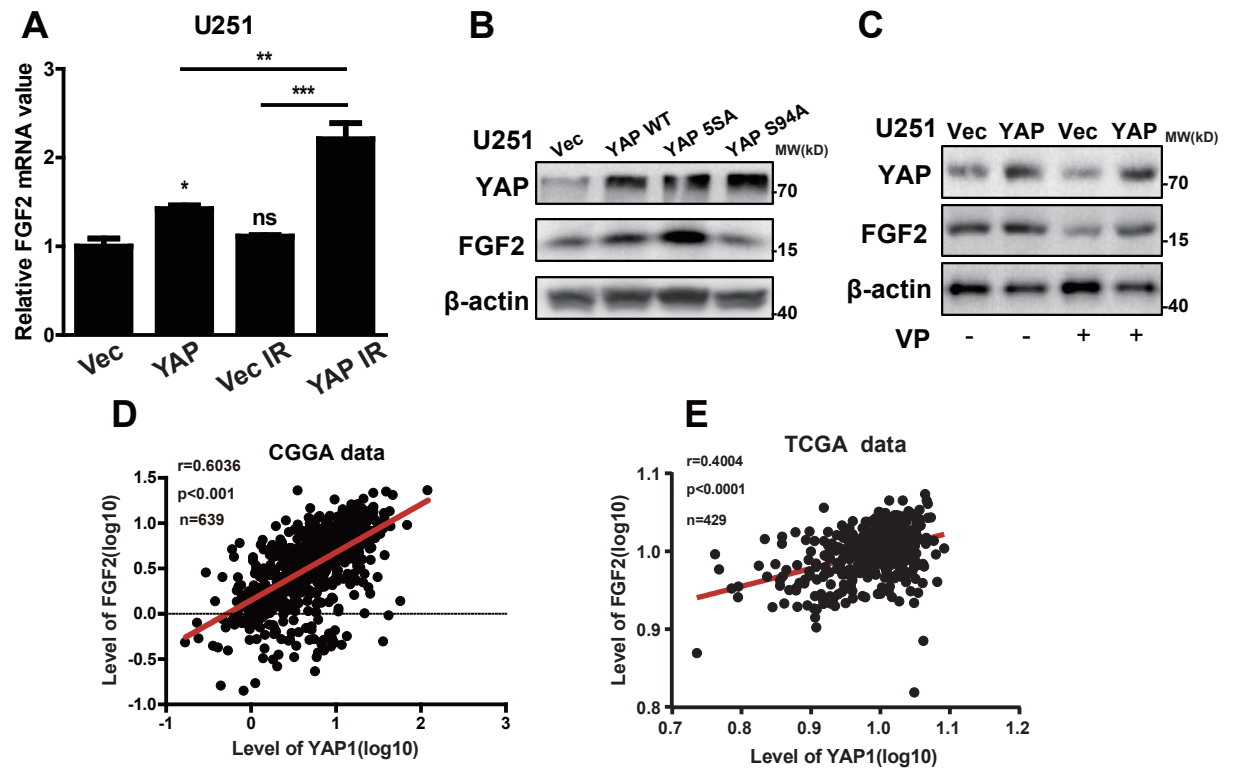

Supplement: Supplementary file 4 — Supplementary Figure 3 [file 41388_2021_1878_MOESM4_ESM.pdf]

sFig.4

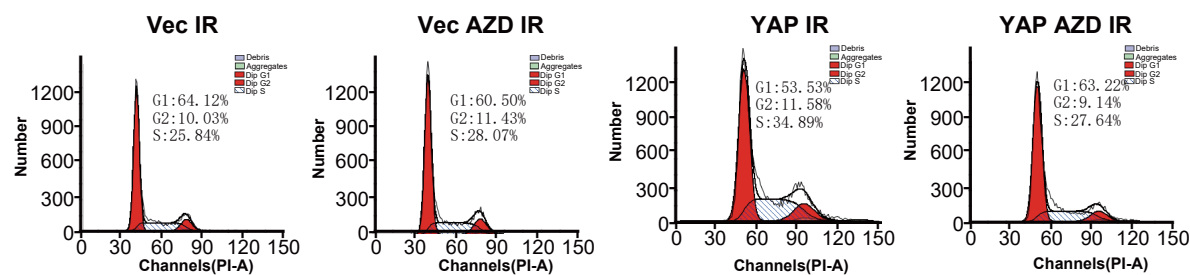

Supplement: Supplementary file 5 — Supplementary Figure 4 [file 41388_2021_1878_MOESM5_ESM.pdf]

sFig.5

A

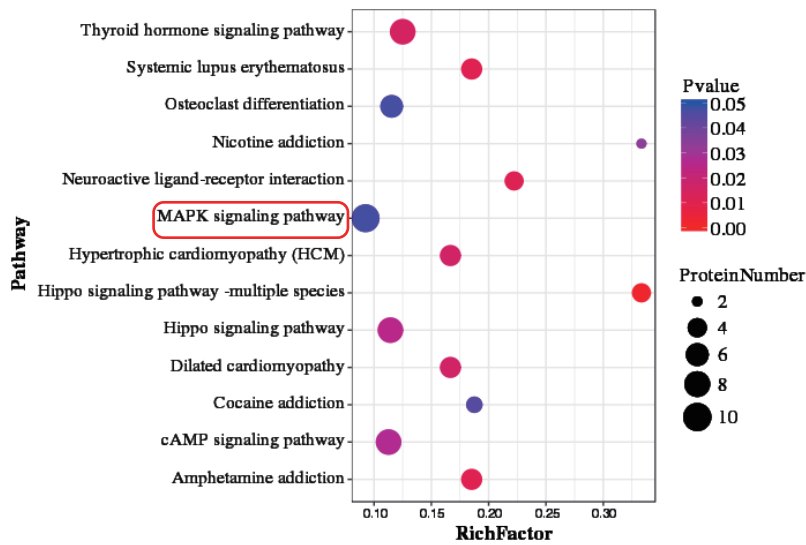

B

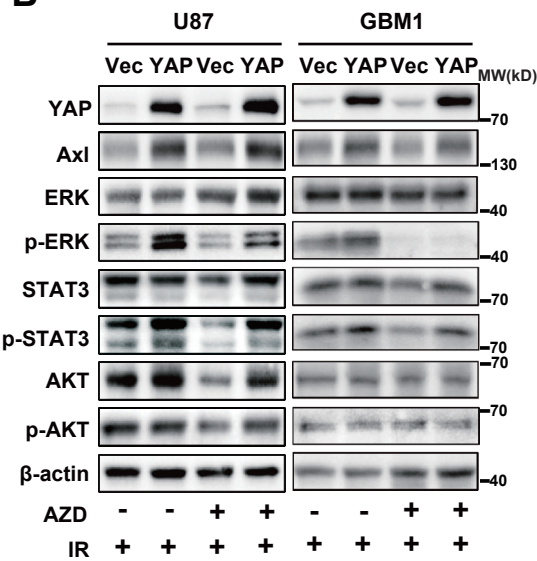

Supplement: Supplementary file 6 — Supplementary Figure 5 [file 41388_2021_1878_MOESM6_ESM.pdf]

sFig.6

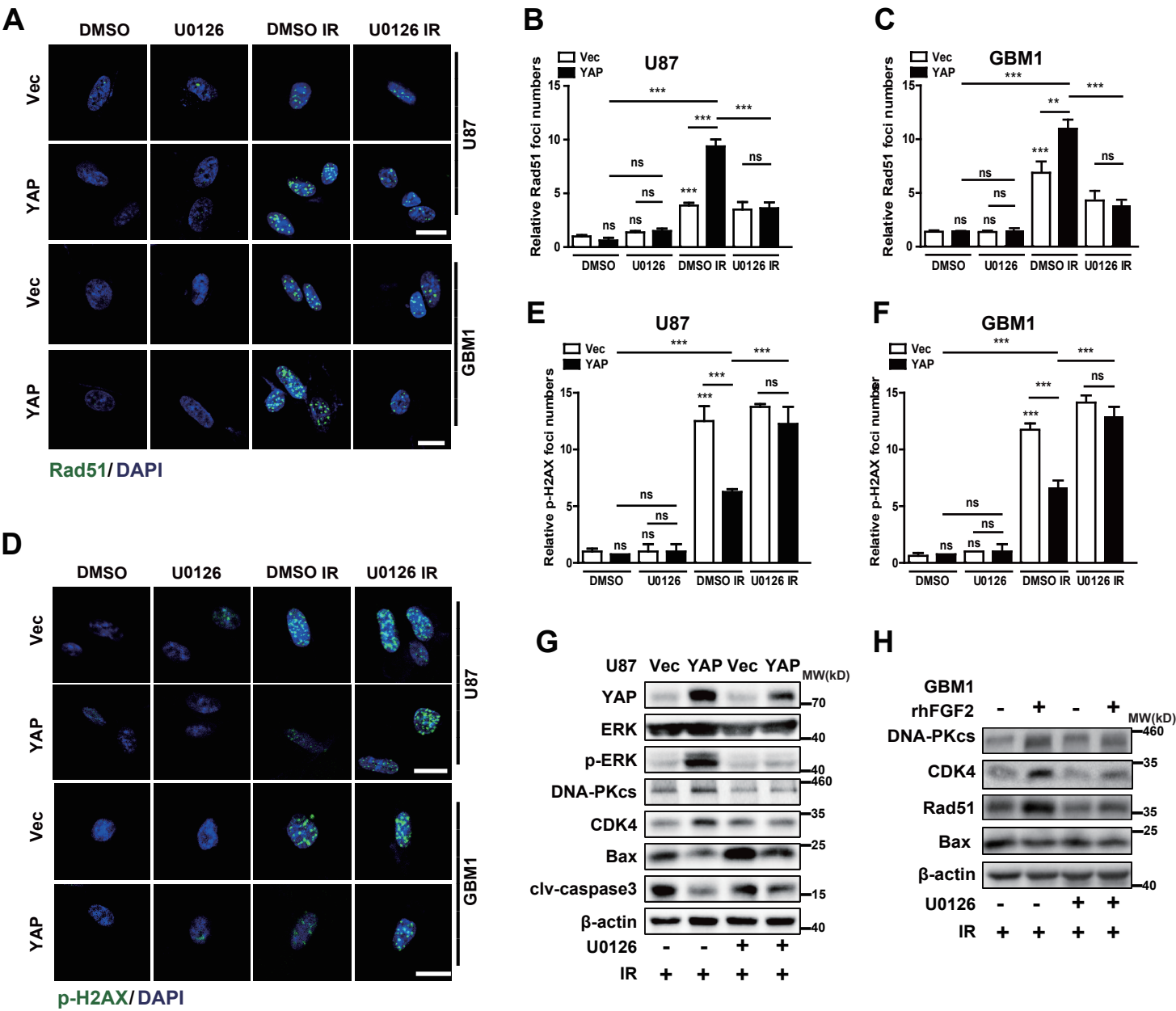

Supplement: Supplementary file 7 — Supplementary Figure 6 [file 41388_2021_1878_MOESM7_ESM.pdf]

**sFig.7**

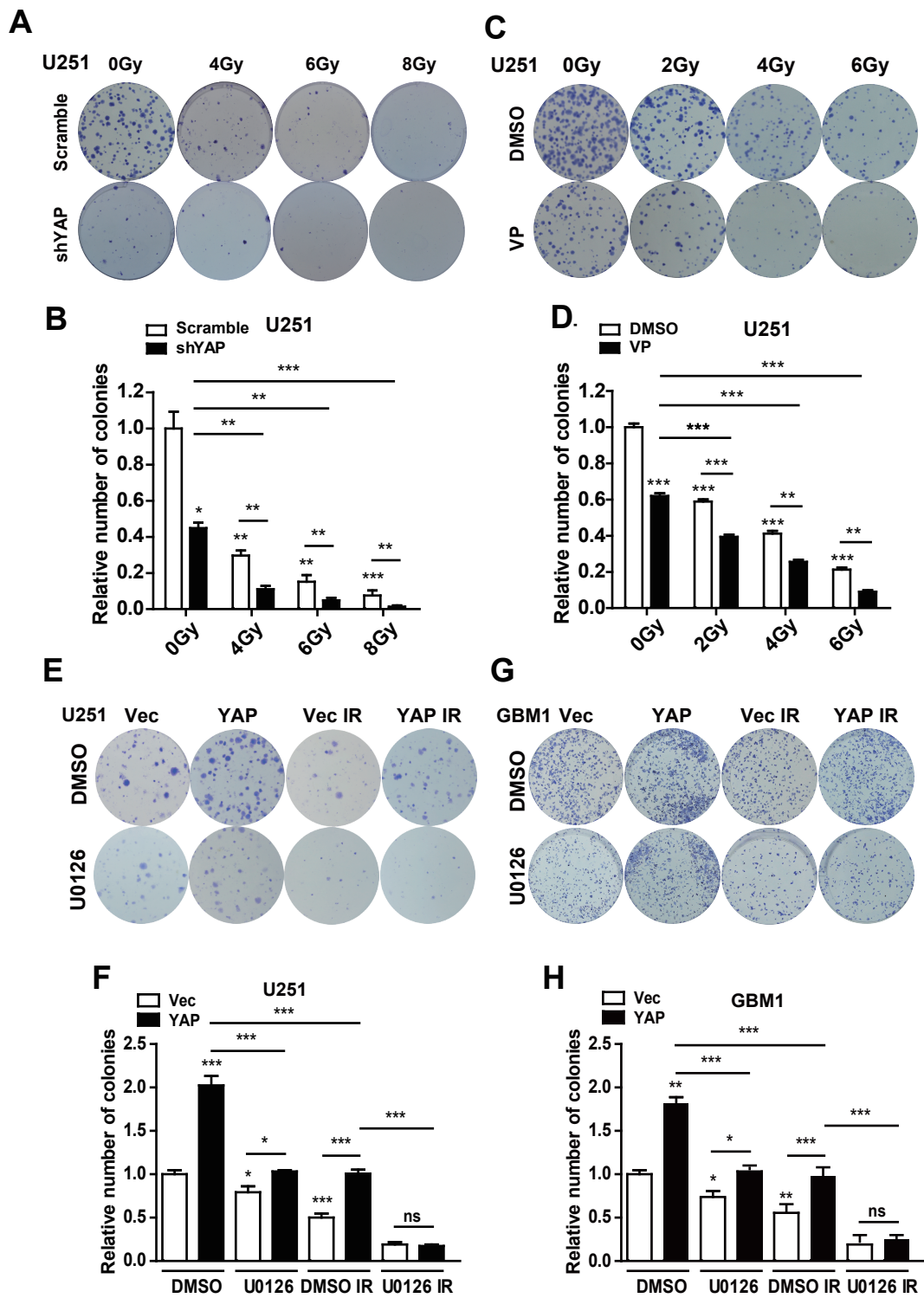

Supplement: Supplementary file 8 — Supplementary Figure 7 [file 41388_2021_1878_MOESM8_ESM.pdf]
